# A GPU-accelerated compute framework for pathogen genomic variant identification to aid genomic epidemiology of infectious disease: a malaria case study

**DOI:** 10.1093/bib/bbac314

**Published:** 2022-08-09

**Authors:** Giovanna Carpi, Lev Gorenstein, Timothy T Harkins, Mehrzad Samadi, Pankaj Vats

**Affiliations:** Department of Biological Sciences, Purdue University, West Lafayette, IN, USA; Purdue Institute for Inflammation, Immunology, & Infectious Disease, Purdue University, West Lafayette, IN, USA; W. Harry Feinstone Department of Molecular Microbiology and Immunology, Johns Hopkins Malaria Research Institute, Johns Hopkins Bloomberg School of Public Health, Baltimore, MD, USA; Rosen Center for Advanced Computing, Purdue University, West Lafayette IN, USA; NVIDIA, 2788 San Tomas, Santa Clara, CA, USA; NVIDIA, 2788 San Tomas, Santa Clara, CA, USA; NVIDIA, 2788 San Tomas, Santa Clara, CA, USA

**Keywords:** pathogen genomics, whole-genome sequencing, variant calling, SNPs, malaria

## Abstract

As recently demonstrated by the COVID-19 pandemic, large-scale pathogen genomic data are crucial to characterize transmission patterns of human infectious diseases. Yet, current methods to process raw sequence data into analysis-ready variants remain slow to scale, hampering rapid surveillance efforts and epidemiological investigations for disease control. Here, we introduce an accelerated, scalable, reproducible, and cost-effective framework for pathogen genomic variant identification and present an evaluation of its performance and accuracy across benchmark datasets of *Plasmodium falciparum* malaria genomes. We demonstrate superior performance of the GPU framework relative to standard pipelines with mean execution time and computational costs reduced by 27× and 4.6×, respectively, while delivering 99.9% accuracy at enhanced reproducibility.

## Background

Pathogen genomic data are a valuable resource for understanding the epidemiology and evolution of infectious diseases. As the current COVID-19 pandemic has demonstrated, a key tool for disease control is the establishment of genomic surveillance programs to monitor local and global patterns of pathogen spread and evolution to guide public health policy decisions and interventions. Advances in next-generation sequencing (NGS) platforms and the rapidly declining sequencing costs have resulted in an unprecedented proliferation of pathogen whole genomes, revolutionizing the field of genomic epidemiology by inferring geographic origins of infections, tracking pathogen transmission and variant spread to aid surveillance and public health response [1–7]. The increasing accessibility of pathogen whole-genome sequencing (WGS) data has spurred the need for robust, rapid, scalable and reproducible bioinformatics pipelines to keep pace with the advances in the NGS market. New scalable compute frameworks are needed to accelerate and accurately process raw sequence reads into analysis-ready variants to speed up and facilitate public health and research communities in the use of pathogen WGS to support surveillance and epidemiological investigations, such as inference of transmission chains, detection of outbreaks and tracking of antimicrobial drug resistance emergence and spread. In turn, this would enable the generation of actionable data within a time frame useful for public health response.

Among human infectious diseases of global importance, malaria is a leading cause of death in children under five years of age in tropical and subtropical regions [8], and halting transmission and spread of antimalaria drug resistance of *Plasmodium falciparum*, the most virulent human malaria parasite, is crucial to reducing the global burden of the disease. Inferring malaria transmission, mapping the spread of antimalaria resistance parasites, developing and evaluating efficacy of vaccines rely on high-quality measurement of natural genetic variation of representative *P. falciparum* WGS data from clinical isolates and population-based surveillance, which is becoming more widely accessible and cheaper [9–17]. Furthermore, *P. falciparum in vitro* evolution and whole genome analysis is increasingly being used to discover the next-generation targets for antimalarial therapeutics [18, 19]. Yet, despite the growth of *P. falciparum* whole genome data, current sequence read mapping algorithms and variant calling tools lack scalability, while studies lack standardization of implemented pipelines. Since the first *P. falciparum* reference genome sequenced in 2002 [20], the number of *P. falciparum* sequenced genomes exceed 7000 [9] and the MalariaGEN Community Project is well on its way to expand sequencing of *Plasmodium* whole genomes. This proliferation of malaria genomic data owns to increase of genomic sequencing capabilities, steady decline in sequencing costs and development of new efficient sample preparation methods to selectively enrich *P. falciparum* whole genomes directly from routinely collected dried blood samples (i.e. hybrid capture, selective whole genome amplification) [14, 21–24]. As the total amount of genomic data is increasing at a rate faster than Moore’s Law for computational processing power, more efficient and scalable compute capacities are required to meet the increased demand for *P. falciparum* WGS processing and variant identification, along with standardized and reproducible bioinformatics pipelines. The limited consensus of standardization in malaria and other pathogen genomic studies is in part due to the fact that conventional variant calling tools (i.e. GATK, DeepVariant [25, 26]) were designed and validated for the measurement of human genomic variation (diploid genomes) rather than microbial haploid genomes [27]. Additionally, standardized genetic variant calling pipelines for pathogens requires the implementation of version controls and truth sets of genetic variants to train data models to measure and assess the performance of these variant calling pipelines. Specifically, for *P. falciparum* genomic studies, new variant calling pipelines need optimization and validation as significant differences in genomic characteristics occur relative to human or other pathogen genomes, including extreme A-T content [20], ploidy issues and low complexity regions.

Existing algorithms and bioinformatics tools developed for conducting analysis-ready variants typically leverage the central processing unit (CPU) environment for data processing [28–31]. However, more recently, new algorithms have been developed that can leverage the computing power of graphics processing unit (GPU) architecture and adopted into high-performance computing workflows as they can outperform CPUs by dramatically reducing computational time when applied to human genomics [32]. To overcome the processing bottlenecks to meet increasing throughput requirements of malaria WGS data and improve reproducibility, we propose NVIDIA Clara Parabricks software (hereafter ‘Parabricks’), a GPU computational framework to accelerate, scale and standardize secondary analysis of *P. falciparum* genome sequencing data. Herein, we optimize and evaluate the performance, sensitivity and precision of Parabricks accelerated mapping and variant calling pipeline against the Burrows-Wheeler Aligner (BWA-MEM) and Genome Analysis Toolkit (GATK), the best practices pipeline using nearly 1000 *P. falciparum* whole genome sequences from the MalariaGEN Community Project [9]. We demonstrate orders of magnitude improvement in the computational time and cost for the GPU-accelerated malaria genomic variant identification pipeline relative to the standard pipeline, while maintaining high accuracy and enhancing reproducibility. This framework can be expanded to any traditional application of *P. falciparum* genomics to exploit the computing power and prevalence of GPUs, and as such has substantial utility within the malaria genomic communities for ensuring scalable, high-quality, reproducible and decentralized analysis-ready variants required for downstream analysis like malaria transmission inferences, antimalaria drug resistance tracking and drug discovery.

## Results

### Parabricks computational workflow overview

**Figure 1:**
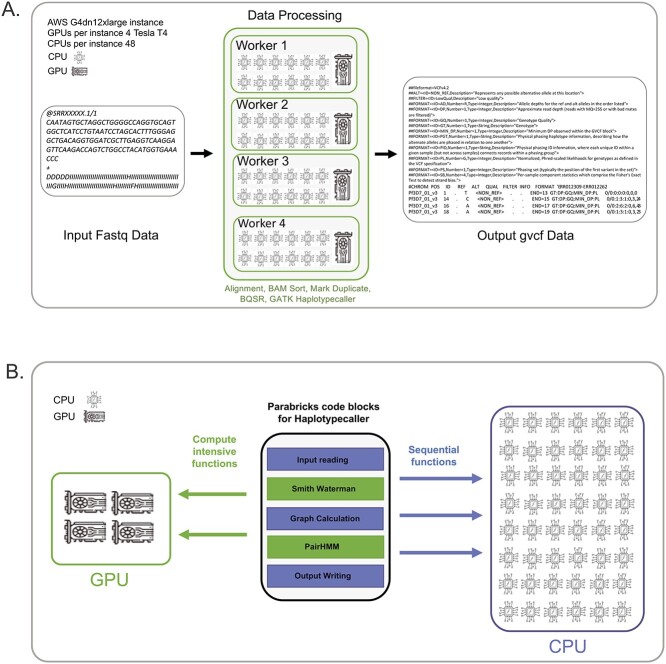
Overview of Parabricks computational workflow. (**A)** The cloud-based Parabricks malaria pipeline presented herein runs on AWS g4dn.12xlarges with 4 Tesla T4 GPUs and 48 CPUs. The pipeline uses fastq files as input and distributes the files into chunks between worker threads for short-read alignment, sorting and marking duplicate reads, calculating BQSR, and finally performing GATK variant calling resulting in output VCF file. (**B)** Work distribution between CPUs and GPUs using Parabricks GPU-accelerated GATK Haplotypecaller for variant calling. The sequential functions for variant calling, such as input reading, graph calculation and output writing are performed on CPU threads, while compute intensive functions, such Smith-Waterman and pair-HMM are performed on GPUs.

Parabricks is a computational workflow optimized to take advantage of GPUs and distribute the inputs among available worker threads on a GPU and CPU hybrid system (Figure 1A). Computationally intensive functions in genomic data analysis, such as Smith-Waterman and Pair-Hidden Markov Model (Pair-HMM) algorithms, are accelerated on GPUs, while sequential functions such as input reading, graph calculation and output writing are run on available CPU threads (Figure 1B). Figure 1 illustrates the Parabricks compute framework ran on AWS g4dn.12xlarge instance with 48 virtual CPUs and 4 Tesla T4 GPUs.

### Pipeline benchmarking and runtime comparison

**Figure 2:**
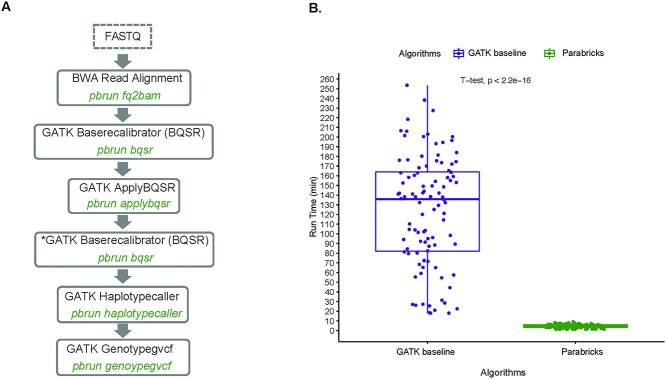
Parabricks variant calling acceleration and scalability. (**A)** Parabricks workflow to perform read mapping and variant calling of malaria *P. falciparum* genomes. The first GATK Baserecalibrator (BQSR) function in the Parabricks pipeline generates a recalibrated bases BAM files, while the second GATK BQSR function, marketed by an asterisks, generate recalibration table outputs to enable the comparison pre- and postrecalibration. (**B)** Runtime comparison between GATK CPU pipeline versus Parabricks GPU-accelerated variant calling pipeline. Boxplots of run time of GATK CPU standard pipeline (purple) and Parabricks GPU pipeline (green) for 100 *P. falciparum* genomes. Bold lines indicate the median value, the boxes span the interquartile range and whiskers extend to the extremes of the sampled values. Parabricks GPU shows a 27× acceleration in execution time relative the GATK CPU standard pipeline (*t*-test, *P*-value <2.2e-16).

Using *P. falciparum* WGS data from six samples with a representative range of BAM file size (median = 2.6 GB, range 0.4–4.7 GB), we estimated the optimal computing configuration to perform benchmarking of Parabricks GPU (Figure 2A) relative to the BWA-MEM and GATK CPU standard pipeline. Tests were run with 1, 4, 8 GPUs, which showed a 2.6×, 2.7× relative speed increase (Table 1, Figure S1). In contrast, test runs with 16, 32, 64 vCPUs showed a 0.9×, 0.8× relative speed decrease, demonstrating that the GPU architecture does not suffer from a diminishing return when increasing allocated processing units compared to CPUs. Based on these estimates (Table 1), we determined that the optimal configuration to benchmark Parabricks pathogen genomic variant identification pipeline is 4 GPUs and 16 vCPUs for BWA-MEM and GATK standard pipeline. Run time comparison based on 100 *P. falciparum* WGS randomly selected samples shows a significant speed increase with a 27× acceleration relative to the GATK CPU standard pipeline (*t*-test, *P*-value <2.2e-16), with a median run time of 5.03 versus 135.73 min per sample (Figure 2B). For both the CPU and GPU pipelines, the run time correlates with the size of the raw input data (Figure 2B). The CPU-based and the Parabricks GPU pipelines exhibit similar ratios of mean runtime to the runtime standard deviation (mean/std) of 124/57 = 2.2 and 4.5/1.9 = 2.4, respectively. The Parabricks GPU is consistently faster than the CPU, and the linear correlation of runtime to input size (data not shown) indicates CPU and GPU both respond similarly to changes in the size of input data for the ranges we ran (median: 2.1 GB, range: 0.02–20.1 GB).

**Table 1:** Benchmarking runtime and cost per *P. falciparum* WGS sample: comparison between Parabricks GPU framework and GATK best practices pipeline

	*aws instances —>*	*g4dn.4xlarge*		*g4dn.12xlarge*		*g4dn.metal*		*m5a.4xlarge*		*m5a.8xlarge*		*m5a.16xlarge*	
Sample	Bam size (GB)	1GPU (min)	Cost ($)	4GPU (min)	Cost $(g4dn.12x)	8GPU (min)	Cost $(g4dn.metal)	16vCPU (min)	Cost $(m5a.4x)	32vCPU (min)	Cost $(m5a.8x)	64vCPU (min)	Cost $(m5a.16x)
ERR015316	1.7	6.56	0.13	3.01	0.20	3.3	0.43	72.39	0.83	75.53	1.74	86.12	3.95
ERR015325	1.8	8.44	0.17	3.36	0.22	3.38	0.44	82.32	0.95	87.03	2.00	92.56	4.24
ERR015328	0.432	2.24	0.04	1.23	0.08	1.56	0.20	28.53	0.33	31.11	0.72	36.21	1.66
ERR015338	4.6	18.59	0.37	7.51	0.49	5.49	0.72	176.5	2.03	194.3	4.47	196.47	9.00
ERR015341	4.7	19.45	0.39	6.44	0.42	6.19	0.81	181.33	2.09	188.57	4.34	205.02	9.40
ERR015365	3.3	15.04	0.30	5.36	0.35	5.04	0.66	152.55	1.75	161.39	3.71	178.23	8.17
		avg cost 1GPU	0.23	avg cost 4 GPU	0.29	avg cost 8 GPU	0.54	avg cost 16 vCPU	1.33	avg cost 32 vCPU	2.83	avg cost 64 vCPU	6.07

Each row represents a *P. falciparum* WGS sample (SRR accession number). Benchmarking was performed on AWS on different configurations. Tests were run with 1, 4, 8 GPUs for the Parabricks pipeline and with 16, 32, 64 vCPUs for the GATK CPU standard pipeline. Tests were run with 1, 4, 8 GPUs, which showed a 2.6×, 2.7× relative speed increase (Although 1 GPU is not supported, it was used to demonstrate the scaling factors; Table 1, Figure S1.) In contrast, test runs with 16, 32, 64 vCPUs showed a 0.9×, 0.8× relative speed decrease. Run cost per sample is reported for each configuration. Average running costs are highlighted in the last row

### GPU-accelerated pathogen genomic variant identification pipeline accuracy

To evaluate the performance of the GPU framework relative to the commonly used read mapping and variant calling tool combination in recovering *P. falciparum* genome-wide variants, we performed read mapping, marked duplicates and variant calling for 979 *P. falciparum* genomes using the BWA-MEM and GATK CPU standard pipeline. We used the GATK CPU output variants as gold standard and truth call set against which to compare the variants called by the Parabricks GPU-accelerated pipeline. The same input dataset was run using the Parabricks GPU *P. falciparum* genomic variant identification pipeline (Figure 2A). Various quality metrics were assessed per genome sample including duplication rates and mean depth coverage (Table S1, Supplementary information, Figures S2 and S3). We measured accuracy in terms of sensitivity (the probability of true variants being identified) and precision (the probability a variant identified by the GPU caller being a true variant). It should be noted that the baseline variant caller in GATK4 is non-deterministic and can generate slightly different results depending upon certain run-time parameters, such as number of threads, so the following differences are consistent with these variations in GATK4 execution. The site-level concordance metrics for the variants identified by the two pipelines are summarized in Table S1. Prior to filtering, the median sensitivity and precision for single nucleotide polymorphisms (SNPs) are 99.97 and 99.95%, respectively. The median sensitivity and precision for short insertion–deletions (InDels) also exceed 99.9%, with values of 99.98 and 99.97%, respectively. The median number of false positive (FP) for SNPs is 33 (range: 0–479) and FP for InDels is 12 (range: 0–359) before filtering. We report an overall high degree of site-level concordance between the GPU pipeline relative to the CPU pipeline with only 0.109 and 0.074% discordant site-level for SNPs and InDels.

**Figure 3:**
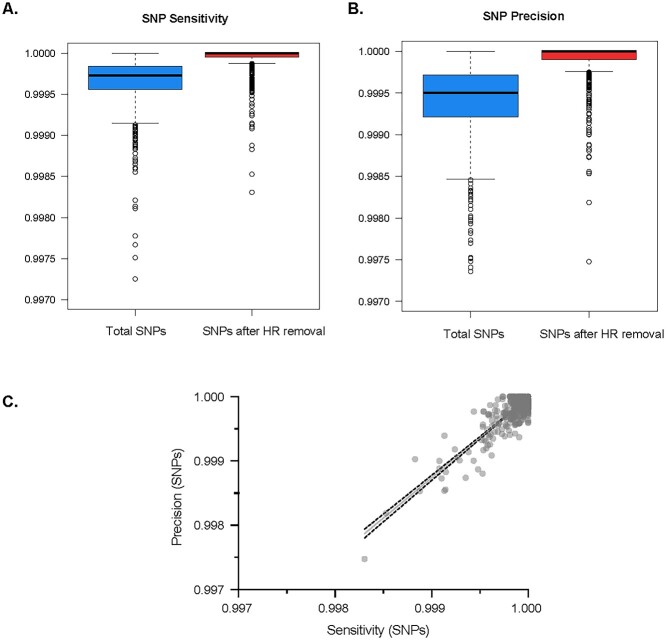
Sensitivity and precision of *P. falciparum* genome-wide SNP identification using Parabricks GPU-accelerated pipeline. Boxplots of (**A**) SNP sensitivity and (**B**) SNP precision after HR removal. Bold lines indicate the median value, the boxes span the interquartile range and whiskers extend to the extremes of the SNP values. (**C**) Scatterplot of SNP sensitivity and precision after HR removal.

**Figure 4:**
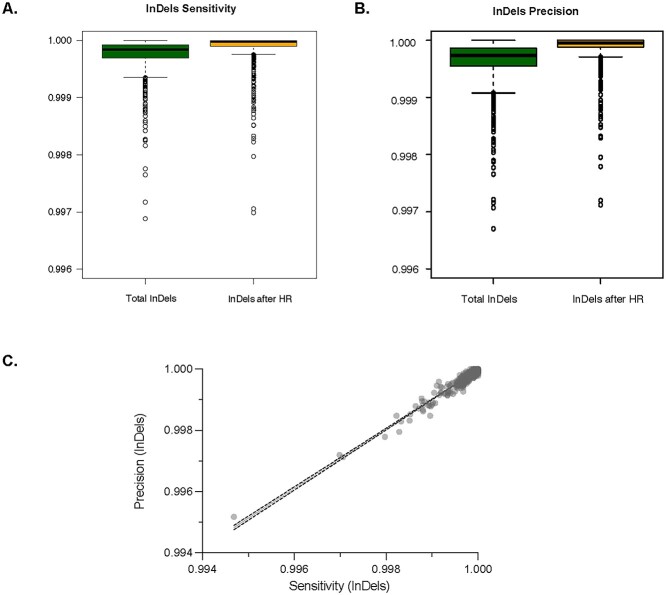
Sensitivity and precision of *P. falciparum* genome-wide InDels identification using Parabricks GPU-accelerated pipeline. Boxplots of (**A**) InDel sensitivity and (**B**) InDel precision after HR removal. Bold lines indicate the median value, the boxes span the interquartile range and whiskers extend to the extremes of the SNP values. (**C**) Scatterplot of InDel sensitivity and precision after HR removal.

Variant calling performance varies across the *P. falciparum* genome and, as expected, worsen in subtelomeric and hypervariable regions (hereafter ‘HR’, see section Methods), where accessibility and unambiguous alignments are limited by repetitive sequences and are generally excluded from *P. falciparum* genomic epidemiology studies [9, 13, 33]. After variants were filtered from HR, we report significant improvements in accuracy in the Parabricks GPU variant calling performance across the *P. falciparum* core genome (20.8 Mb; 90%). The median sensitivity and precision for SNPs are 100 and 100%, respectively, and as expected are significantly improved over the accuracy prior filtering out variants in the HR (Figure 3A, B Wilcoxon, *P* < 0.0001). Similarly, the median sensitivity and precision for InDels are 99.99 and 99.99%, respectively, with a resulting accuracy significantly improved over the accuracy prior filtering (Figure 4A, B, Wilcoxon, *P* < 0.0001). Notably, after HR variants were filtered, both pipelines achieved high genotype concordance (degree of agreement between genotype data) (Table S3), with an overall genotype concordance of 99.9971% for SNPs and 99.9915% for InDels. This demonstrates that SNP and InDel alleles are highly concordant between the CPU and GPU pipelines, with InDels having slightly higher discordant genotypes compared to SNPs, as should be expected. This demonstrates that Parabricks GPU *P. falciparum* variant calling pipeline is functionally equivalent to the GATK CPU standard tools, maintaining an accuracy greater than 99.9 while mean execution time is significantly accelerated (Figure 2B).

Performing sequence read mapping and variant calling for *P. falciparum* WGS samples that have multi-fastq files is laborious, time consuming and prone to errors. Parabricks GPU *P. falciparum* variant identification pipeline is optimized to take as input multiple paired fastq files from the same sample in a single command line, to perform mapping, sorting and mark duplicates and merging to create sample-level BAM files. In the analyzed dataset, 119 out of the 979 *P. falciparum* genome samples had multi-fastq files (range: 2 to 12 multi-fastq paired files) and were run using this function. This optimized function in Parabricks, which is not available in the BWA-MEM CPU standard pipeline, simplifies the pathogen genomic mapping and variant identification pipeline and enhances reproducibility, while maintaining reduced execution time.

While sequencing costs of pathogen WGS have reduced drastically, computational costs to perform mapping and variant calling remain relatively high [34]. We compared the average running cost per *P. falciparum* genome of the GPU framework relative to the BWA-MEM and GATK CPU standard pipeline in recovering *P. falciparum* genome-wide variants. We demonstrate up to fivefold reduced running cost for the GPU framework against the BWA-MEM and GATK CPU baseline using the optimal hardware configurations (4 GPU versus 16 vCPU) (Table 1). Given the average computing cost per *P. falciparum* genome using the GPU-accelerated framework of $0.29 on AWS, we determine that computing 1000 P*. falciparum* genomes from raw fastq to analysis-ready variants on 4 GPU costs approximately $290. In comparison, given the average computing cost per *P. falciparum* genome using the standard BWA-MEM and GATK CPU pipeline of $ 1.33 on 16 vCPU, for the same number of *P. falciparum* genomes the cost is $1330. This demonstrates that the proposed GPU-accelerated pipeline for *P. falciparum* genome-wide variant identification is not only fast and highly scalable but also considerably minimizes bioinformatics costs per sample rendering large-scale *P. falciparum* genomic studies relatively inexpensive and feasible to the malaria research community and to surveillance programs.

### Comparison between cloud-based ecosystem and in-house HPC for reproducibly purpose

A subset of 100 samples was randomly selected from the full 979 *P. falciparum* genomes set and analyzed using the malaria Parabricks GPU genomic variant identification pipeline on the Purdue University’s Gilbreth HPC cluster for a reproducibility study. No modifications were implemented to the pipeline settings or driving scrips except for site-specific storage paths and scheduler parameters. Produced pipeline results were compared to corresponding matching samples from the AWS cloud-based runs. We assessed the accuracy in terms of sensitivity and precision of identified genome-wide variants in the 100 *P. falciparum* genomes run on Gilbert HPC versus AWS using the GATK concordance function. The results are summarized in Table S2. The median sensitivity and precision for SNPs and InDels for the 100 *P. falciparum* genomes run on Gilbert HPC versus AWS are 100 and 100%, respectively. This comparison demonstrates that the malaria Parabricks GPU genomic variant identification pipeline can be run on cloud-based system and on an in-house HPC that have significantly different hardware (Tesla T4GPUs versus HPC P100) and still produce the same qualitative and quantitative variant results, as should be expected by a reproducible and deterministic software, while maintaining acceleration and scalability factors.

## Discussion and conclusion

The utility of pathogen genomic data for surveillance and control of infectious diseases is evidenced by the COVID-19 pandemic, for which viral WGS analysis has greatly informed patterns of variant spread, virus evolution and vaccine efficacy [3–6]. The increasing affordability of NGS for WGS has revolutionized pathogen genomic surveillance programs by providing inexpensive sequencing data to monitor transmission and evolution of malaria, COVID-19, and other pathogens in clinical and public health settings. These leaps in pathogen genomics and sequencing technologies promise to enable corresponding advances in biomedicine and genomic epidemiology of infection diseases for research discovery purpose and for improving public health efforts. Although, many obstacles and bottlenecks remain in the entire process from sample collection to end analysis, a critical step in the NGS workflow is the bioinformatics analysis for variant identification in large-scale pathogen genome data sets, which is hampered by the drastic increase in sequencing data production at continued reduced costs. To overcome this computational challenge and enable the full power of these large pathogen WGS data sets, we must take advantage of the computing power and scalability of the GPU architecture and computing workflows, which have shown to outperform traditional CPU bioinformatics pipeline for human genomics and in critical care settings [32, 35]. In this study, we demonstrate that the GPU-accelerated Parabricks pipeline for *P. falciparum* genomic variant identification exhibits a large performance advantage over the conventional pipeline by improving speed, scalability and reproducibility. Our comparative analysis of Parabricks GPU accuracy relative to the standard GATK CPU pipeline demonstrates that Parabricks GPU performs comparably to previous methods, maintaining high accuracy with >99.9% for SNP and InDel detection, while significantly reducing execution time by 27-fold and computational costs by fivefold. The ability to generate analysis-ready variant outputs in less than 5 min with greater than 99.9% accuracy for the high confidence genomic regions of *P. falciparum* at lower costs, remarkably reduces the computational bottleneck that most malaria genomic epidemiology studies and surveillance programs currently face. This means that a malaria WGS workflow for variant detection that normally takes 2–3 hrs. Per sample on a CPU-only node can be processed on a GPU-enabled Parabricks pipeline in less than 5 min. Notably, this speed gain becomes useful to the public health and research communities to rapidly generate insights into their samples as WGS are generated in the lab, from the mining of antimalaria drug resistance profiles to compute and recomputing comparative analyses of malaria genomes from temporally and spatially relevant data set as they are generated or become available in public data repositories.

*P. falciparum* genomic studies from natural populations have increasingly being used to guide our understanding of malaria epidemiology and transmission, for example by assessing population connectivity between sub-populations, and the efficacy of parasite interventions [9, 11, 13, 15, 16, 36], and will continue to grow as the World Health Organization (WHO) has called for reinvigorated commitment to malaria elimination. *P. falciparum* WGS is an essential tool and remains the gold standard for identifying and cataloguing new genetic variants relevant for surveillance. The malaria Parabricks GPU variant identification pipeline provides additional and practical advantages to these efforts: first, its ease-of-use and unified analytic framework enables users with limited bioinformatics expertise to carry out scalable, reproducible analysis-ready variants; second, its suitability for AWS cloud deployment facilitates access to users with limited onsite computational resources at better cost efficiency due to the inherent elasticity of cloud computing. Thus, the efficiency, scalability and ease-of-use of the established framework renders population genomic studies of *P. falciparum* economically more accessible, enabling efforts to decentralize malaria WGS and computational analysis to endemic countries [10, 37]. We note a potential limitation to our study, due to the lack of highly confident gold standard variant call datasets, we did not measure performance of the pipeline in identifying low-frequency variants originating from the minor strain in mixed infections, which are clinically and epidemiologically relevant and can provide additional information for malaria transmission intensity inferences [10, 38]. However, our results show promise for these applications given the accuracy in SNP detection relative to standard methods, while previous studies have shown that downstream analytical methods for deconvolution of mixed infections from *P. falciparum* WGS from short reads are rather limited by number of strains, low genome coverage and reference panels used [39, 40]. This suggests that generating high-quality WGS remain critical and can reduce the possible bias in mixed infection estimation in this study system.

While here we focus on the GPU-accelerated compute framework applied to *P. falciparum* genomics as a case study given the increased interest and application of malaria genomics to support global control and elimination efforts, we propose that Parabricks pipelines can be widely applied in similar way to other sequenced human pathogens, including other protozoan and bacterial genomes for which large-scale WGS projects are available or under way (for example *Mycobacterium tuberculosis* [41]), and as such has substantial utility within the pathogen genomic community for studies of genetic variation and genomic epidemiology.

Although we demonstrated that the GPU-accelerated compute framework for pathogen genomic variant identification yields improvements in computational speed, scaling and reproducibility, this is only a first step, and benefits will only become greater with continued improvements of GPU based frameworks for downstream WGS analysis. For example, first, improvements in usability will improve the accessibility of WGS analyses; second, extension of Parabricks to machine learning approaches for further execution improvement; third, comparison with a more extensive suite of bioinformatics pipelines to improve community confidence in GPU-accelerated frameworks applied to large-scale pathogen genomic studies.

## Material and methods

### *P. falciparum* WGS benchmarking dataset acquisition and preparation

Throughout this study, we used *P. falciparum* 3D7 v3.1 as a reference genome (ftp://ftp.sanger.ac.uk/pub/project/pathogens/gff3/2015-08/Pfalciparum.genome.fasta.gz), which is an African parasite clone isolated in the Netherlands and is routinely used as a reference for *P*. *falciparum* malaria genomic studies. For the comparison of the two pipelines, we used publicly available *P. falciparum* genomic data from the Pf3k MalariaGEN *P. falciparum* Community Project (http://www.malariagen.net/projects/parasite/pf) [9]. Raw fastq data for all Pf3k *P. falciparum* samples were downloaded from SRA using pysradb (https://github.com/saketkc/pysradb) [42]. We randomly selected 50% of *P. falciparum* samples from 10 countries for a total of 1000 *P. falciparum* samples, and excluded any samples with only single-end reads and with the same SRR values present in multiple samples. This resulted in 979 unique *P. falciparum* samples with genomic data from 10 countries. SRR accession numbers are provided in the Supplementary Table S1.

### Read mapping and GATK variant calling

To establish the baseline performance, the raw paired-end reads for 979 *P. falciparum* samples were mapped to the *P. falciparum* 3D7 reference genome using BWA-MEM v0.7.15 [28]. Aligned reads were sorted and duplicates marked using GATK v4.1.0.0. The GATK v4.1.0.0 pipeline was applied following best practices (https://software.broadinstitute.org/gatk/best-practices) to establish the baseline performance of each call set generated from 979 *P. falciparum* genomes. For *P. falciparum* genome samples that had multi-fastq paired files (119 out of 979 samples), each paired-end reads were mapped, sorted and duplicates marked prior BAM files were merged to create sample-level BAM files. GATK Base quality Score Recalibration was applied using default parameters and using variants from the *P. falciparum* crosses 1.0 release as a set of known sites (ftp://www.malariagen.net/data/pf-crosses-1.0) [33, 43]. We used GATK HaplotypeCaller in GVCF mode to call single-sample variants (*ploidy* 2 and *standard-min-confidence-threshold for calling* = 30), followed by GenotypeGVCFs to genotype the cohort. Variants were examined and summarized before and after filtering out variants in sub telomeric and hypervariable regions (ftp://ngs.sanger.ac.uk/production/malaria/pf-crosses/1.0/regions-20130225.onebased.txt).

### GPU-accelerated genomic variant identification pipeline

We used and validated Parabricks Suites v3.2.0.1, which enables GPU-accelerated GATK along with other third-party tools, such as BWA-MEM, to achieve acceleration of *P. falciparum* variant identification pipeline over the conventional CPU pipeline. Parabricks is built to optimize acceleration, accuracy and scalability by keeping the underlying GATK tools the same. We followed the Parabricks *P. falciparum* malaria pipeline illustrated in Figure 2A and applied the pipeline to the same input data set of 979 *P. falciparum* genomes used in the GATK CPU standard pipeline. Documentation and the implemented code can be found at https://github.com/giocarpi/malaria-parabricks-pipeline. GATK’s Concordance tool was utilized to evaluate site-level concordance of the Variant Call Format (VCF) from Parabricks against the VCF GATK CPU output variants as the truth set, for each of the *P. falciparum* WGS sample to assess the degree of agreement of position matching of detected variants. Next, we computed genotype concordance for each *P. falciparum* WGS sample for each of the two call sets after filtering out variants in HRs as described above using the GATK’s Genotype Concordance tool to assess the degree of agreement between genotype data (with GATK CPU being considered the truth (or reference) the Parabricks GPU being the call). All GATK applications used within this study were performed on GATK version 4.1.0.0. A 90 day’s trial license for the Parabricks GPU-accelerated software is available at https://www.nvidia.com/en-us/clara/genomics/.

### Benchmarking of GATK and Parabricks variant calling pipeline for *P. falciparum*

While variant identification pipelines for human genomes are often benchmarked upon diploid human genomic ‘truth sets,’ variants identified and confirmed by multiple sequencing technologies and bioinformatic pipelines, and further validated by family pedigrees [44], comparable genomic variant truth sets for *P. falciparum* and for even other human pathogens are limited [33, 45]. Here, we used the GATK CPU output variants relative to 3D7 reference genome as gold standard and truth call set against which to compare the variant calls by the Parabricks GPU-accelerated pipeline. True positives (TP) are defined as the variant calls called by GATK CPU and Parabricks GPU, FP are the calls only identified by Parabricks GPU and false negatives are the variant calls identified by GATK but missed by Parabricks GPU. We measured accuracy in terms of sensitivity (the probability of true variants identified) and precision (the probability a variant identified by the GPU caller is indeed a true variant). Sensitivity is calculated as TP/(TP + FN) and Precision is calculated as TP/(TP + FP)).

### Computing environment and resources

Amazon could (Amazon Web Service, AWS) and different hardware configurations were used to perform benchmarking for runtime and costs. We used the same sample input data of *P. falciparum* genomes to compare turnaround times and computational costs on 1, 4 and 8 GPU nodes, although 1 GPU not supported was used to demonstrate the scaling factors for the Parabricks pipeline against 16, 32 and 64 vCPU nodes for the GATK CPU standard pipeline. Reproducibility runs were performed on Purdue University’s Gilbreth HPC cluster (https://www.rcac.purdue.edu/compute/gilbreth/) using Gilbreth-B nodes with 192 GB of RAM, 2x12-core Intel Xeon Gold 6126 CPUs and 2 NVIDIA Tesla P100 cards per node.

**Key Points**

- We established an accelerated, scalable, reproducible and cost-effective compute framework to accelerate raw sequence reads into analysis-ready variants to speed up pathogen surveillance efforts and epidemiological investigations.
- We evaluate the performance and accuracy of the GPU-accelerated pipeline against GATK Best Practices using 1000 *Plasmodium falciparum* malaria genomes.
- The GPU-accelerated compute framework for malaria genomic variant identification outperforms the standard pipeline by significantly reducing computational time and costs, 27× and 5×, respectively, while achieving >99.9% accuracy.
- This unified and easy-to-use GPU analytic framework is a reproducible and powerful tool to support various applications in malaria genomics and can be applied in similar way to other human pathogens.

## Supplementary Material

GPU_Framework_PathogenGenomicVariantIdentification_SI_revised_bbac314

Table_S1_bbac314

Table_S2_bbac314

Table_S3_bbac314

## Data Availability

Parabricks malaria pipeline and codes are available in the GitHub repository at (https://github.com/giocarpi/malaria-parabricks-pipeline).

## Author Biographies

**Giovanna Carpi** is an adjunct faculty in the Department of Biological Sciences, Purdue University. Her main research focuses on the application of novel sequencing and bioinformatics methods to aid the interpretation of large-scale pathogen genomic and metagenomic datasets generated in clinical and public health settings to understand infectious disease transmission and evolution.

**Lev Gorenstein** is a senior computational scientist in the Rosen Center for Advanced Computing at Purdue University, specializing in computational and data-management workflows, specialized software support, high-performance computing training and outreach.

**Timothy T Harkins** is a product manager at Nvidia. He has vast experience in developing and commercializing new technologies and products for the genomics industry. He has nearly 70 peer reviewed articles highlighting the applications supported by these technologies.

**Mehrzad Samadi** is an engineering manager at Nvidia. His research interest is in software optimizing and accelerating genomics application for GPUs along with heterogeneous computing and machine learning. He has over 10 years’ experience in high-performance computing and over 20 peer reviewed publications.

**Pankaj Vats** is a Sr. Bioinformatics Scientist at Nvidia, his research focuses on precision medicine along with developing and applying new bioinformatics algorithms on next-generation sequencing data. Most of his efforts centered toward utilizing bioinformatics methods in clinical sequencing, cancer, and population genomics to understand the underneath mechanism of the disease condition.
