# Supplementary material for: A GPU-accelerated compute framework for pathogen genomic variant identification to aid genomic epidemiology of infectious disease: a malaria case study
Source: Brief Bioinform. 2022 Aug 9;23(5):bbac314. doi: 10.1093/bib/bbac314 (PMC9487672; doi:10.1093/bib/bbac314)
Supplement: GPU_Framework_PathogenGenomicVariantIdentification_SI_revised_bbac314 [file gpu_framework_pathogengenomicvariantidentification_si_revised_bbac314.docx]

**Supplementary Information for**

**A GPU accelerated compute framework for pathogen genomic variant identification to aid genomic epidemiology of infectious disease: a malaria case study**

Giovanna Carpi ^1,2,3 #^, Lev Gorenstein ^4^, Timothy T. Harkins ^5^, Mehrzad Samadi ^5^, Pankaj Vats ^5, #^

^1^ Department of Biological Sciences, Purdue University, West Lafayette, IN, USA

^2^ Purdue Institute for Inflammation, Immunology, & Infectious Disease, Purdue University, West Lafayette, IN, USA

^3^ W. Harry Feinstone Department of Molecular Microbiology and Immunology, Johns Hopkins Malaria Research Institute, Johns Hopkins Bloomberg School of Public Health, Baltimore, MD, USA

4 Rosen Center for Advanced Computing , Purdue University, West Lafayette IN, USA

^5^ NVIDIA, 2788 San Tomas, Santa Clara, CA, USA

^#^ Corresponding Author

Email addresses:

Giovanna Carpi: [gcarpi@purdue.edu](http://gcarpi@purdue.edu)

Lev Gorenstein: [lev@purdue.edu](http://lev@purdue.edu)

Timothy Harkins: [tharkins@nvidia.com](http://tharkins@nvidia.com)

Mehrzad Samadi: [msamadi@nvidia.com](http://msamadi@nvidia.com)

Pankaj Vats: [pvats@nvidia.com](mailto:pvats@nvidia.com)

**SI Appendix includes:**

- Tables S1,S2 and S3 (provided separately as additional files in Excel format)
- Figures S1 to S4

**Supplementary Tables**

**Supplementary Table S1*.*** *P. falciparum* WGS Mapping and variant calling statistics.

**Supplementary Table S2**. Reproducibility of the GPU accelerated malaria genomic variant identification pipeline on AWS cloud and Purdue HPC cluster for 100 samples.

**Supplementary Table S3*.*** Genotype concordance for each of the 979 *P. falciparum* WGS sample between the two call sets computing using GATK’s Genotype Concordance tool to assess the degree of agreement between genotype data (with GATK CPU being considered the truth set and the Parabricks GPU being the call.

**Supplementary Figures**

**Figure S1. Comparison of run time.** Read mapping and variant calling runtime of Parabricks GPU accelerated pipeline (green shade colors) vs BWA-MEM and GATK4 CPU pipeline (purple shade colors). Benchmarking was performed on three different computing configurations for GPUs and CPUs on AWS using six *P. falciparum* genome samples. The y-axis represents run time expressed in minutes.

**Figure S2. Duplication rates across the 979 *P. falciparum* analyzed genomes using the GPU accelerated variant identification pipeline (Parabricks).** The y-axis represents the duplicate rates at the sample-level BAM file. The x-axis corresponds the *P. falciparum* genome samples (SRR accession number). The median duplicate rate across the samples is 1.43% (range: 0.05 -62.5%).


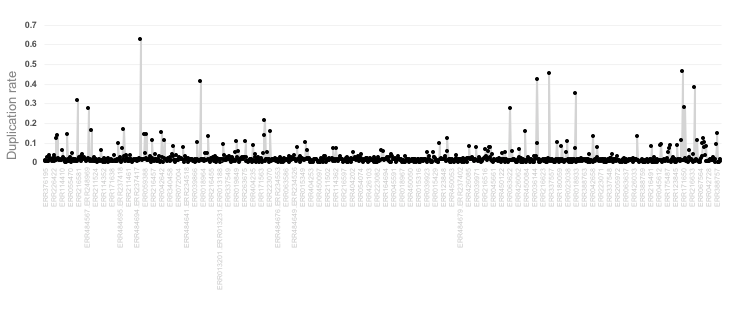
**Figure S3. Mean depth coverage across the 979 *P. falciparum* analyzed genomes using the GPU accelerated variant identification pipeline (Parabricks).** The y-axis represents *P. falciparum* mean genome coverage across the analyzed samples. The x-axis corresponds the *P. falciparum* genome samples (SRR accession number). The median genome coverage was 61X (range: 0.05-232X). Red dotted line highlights samples with mean genome coverage <30X.


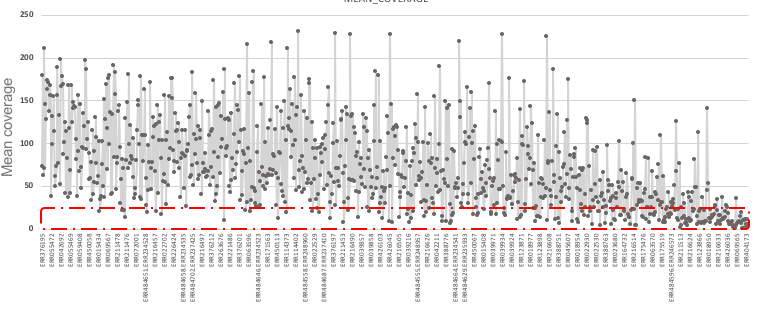


**Figure S4. Total variants identified prior and after hypervariable regions (HR) removal across 979 *P. falciparum* genomes using the GPU accelerated variant identification pipeline.** The y-axes represent the total number of SNPs (above) and the total number of InDels (below). The x-axis corresponds the *P. falciparum* genome samples (SRR accession number). Blue color illustrates the total variants (SNPs or InDels) prior filtering out hypervariable regions (HR). Orange color denotes the total variants (SNPs or InDels) after filtering.

**
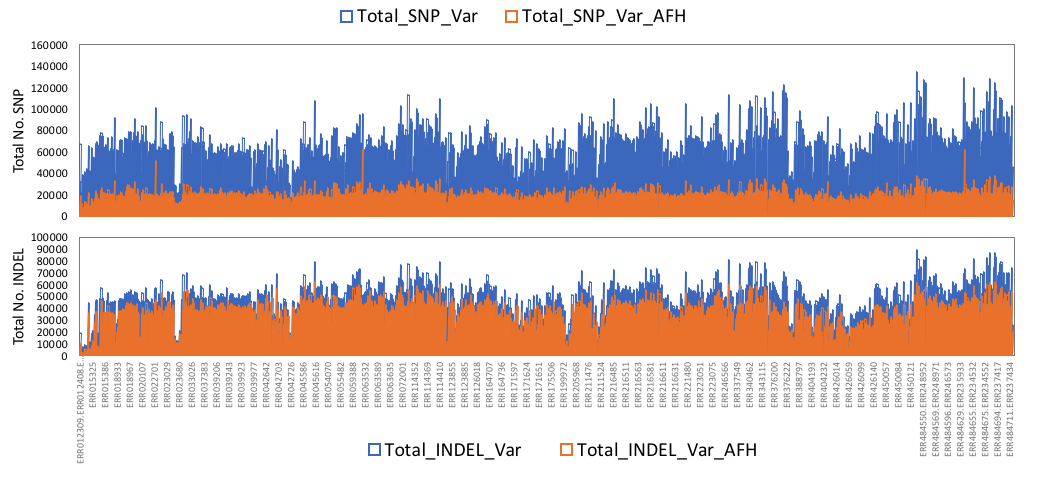
**
